# Case report: Novel NUS1 variant in a Chinese patient with tremors and intellectual disability

**DOI:** 10.3389/fgene.2024.1373448

**Published:** 2024-04-09

**Authors:** Ruolin Li, Jiayi Yang, Jinfeng Ma, Aimei Zhang, Hongfang Li

**Affiliations:** ^1^ Department of Neurology, Affiliated Hospital of Jining Medical University, Jining Medical University, Jining, China; ^2^ Clinical Medical College, Jining Medical University, Jining, China

**Keywords:** amino acid substitution, case report, exome sequencing, intellectual disability, epilepsy, tremor, undecaprenyl pyrophosphate synthetase, young adult

## Abstract

**Introduction:**

Nuclear undecaprenyl pyrophosphate synthase 1 (NUS1) gene variants are associated with a range of phenotypes, including epilepsy, intellectual disability, cerebellar ataxia, Parkinson’s disease, dystonia, and congenital disorders of glycosylation. Additionally, cases describing genotypes and clinical features are rare.

**Case Presentation:**

Herein, we report the case of a 23-year-old Chinese female patient who presented with tremors, intellectual disability, and epilepsy. A history of carbon monoxide exposure, brain trauma, or encephalitis was not present in this case. Trio whole-exome sequencing analysis revealed a *de novo* pathogenic variant of c.750del in exon 4, leading to p.Leu251* amino acid substitution. Genetic analysis failed to identify the identical mutations in the remaining family members who underwent screening. The patient was diagnosed with a rare congenital disease, “congenital glycosylation disorder, type 1aa, autosomal dominant, type 55, with seizures (MRD-55).”

**Conclusion:**

We provide further evidence for the role of variants in *NUS1* in the development of tremors, epilepsy, and intellectual disabilities. These findings expand our understanding of the clinical phenotypes of *NUS1* variants.

## 1 Introduction

Nuclear undecaprenyl pyrophosphate synthase 1 (*NUS1*) gene encodes the precursor of the N-terminal membrane of the Nogo-B receptor (NgBR) (Grabińska et al., 2017; Den et al., 2019; Riboldi et al., 2022), which acts as an essential subunit of the dehydrodolichyl diphosphate synthase (DHDDS) complex and plays a crucial role in maintaining glycosylation in mammals (Harrison et al., 2011; Park et al., 2014; Giladi et al., 2022).

The pathogenic variants of *NUS1* were first identified in a family with a congenital disorder of glycosylation (CDG) (Park et al., 2014; Jaeken and Péanne, 2017). To date, 29 pathogenic or likely pathogenic mutations of *NUS1* have been reported worldwide, 18 of which have traceable clinical phenotypes (according to the Human Mutation database) (Hu et al., 2023). The spectrum of phenotypes includes epilepsy, cerebellar ataxia, tremors, cortical myoclonus, intellectual disability, and psychomotor retardation (Ji et al., 2023). These variants are associated with dystonia, Parkinson’s disease, developmental and epileptic encephalopathy, and CDG (Monfrini et al., 2022). Therefore, different pathogenic variants may cause various diseases. Furthermore, cases describing genotypes and detailed clinical features are limited. Here, we report a case of a patient with a novel *de novo* pathogenic variant of the *NUS1* gene (c.750del, p.Leu251*) who presented with tremors, epilepsy, and intellectual disability.

## 2 Case description

Our patient, a 23-year-old woman, was the second child born to nonconsanguineous healthy parents without any family history of intellectual disability or epilepsy. Her older sister is healthy. She was born spontaneously at full term without dystocia and had no history of carbon monoxide exposure, brain trauma, or encephalitis.

At the age of 6 years, it was noticed that she was intellectual disability, although her physical development (weight and height) was normal. She could walk and speak, and was not receive any medical intervention. At the age of 8 years, she presented with involuntary shaking and tremors of the head and neck, which spread to all her limbs, and were accompanied by an involuntary nod. The symptoms worsened during mood fluctuations. During this period, the patient was conscious with no binocular upward gaze, perioral bruising, or limb stiffness. She underwent brain magnetic resonance imaging (MRI) and electroencephalography (EEG). Brain MRI showed a left choroidal cyst and compression of the hippocampus.

On the basis of extensive slow waves of two–7 Hz, spike waves, sharp waves, and sharp-and-slow waves were observed in the bilateral temporal, parietal, and occipital lobes. Routine blood tests revealed no significant abnormalities. The possibility of epilepsy could not be excluded. The patient was treated with levetiracetam (500 mg, twice daily) and sodium valproate (500 mg, twice daily), which resulted in a slight improvement in the limb tremors. During this period, the patient experienced convulsions four times during quiet sleep; however, her family was unable to describe the symptoms in detail. Subsequently, it was decided to maintain the patient’s current treatment with only a dose adjustment.

**FIGURE 1:**
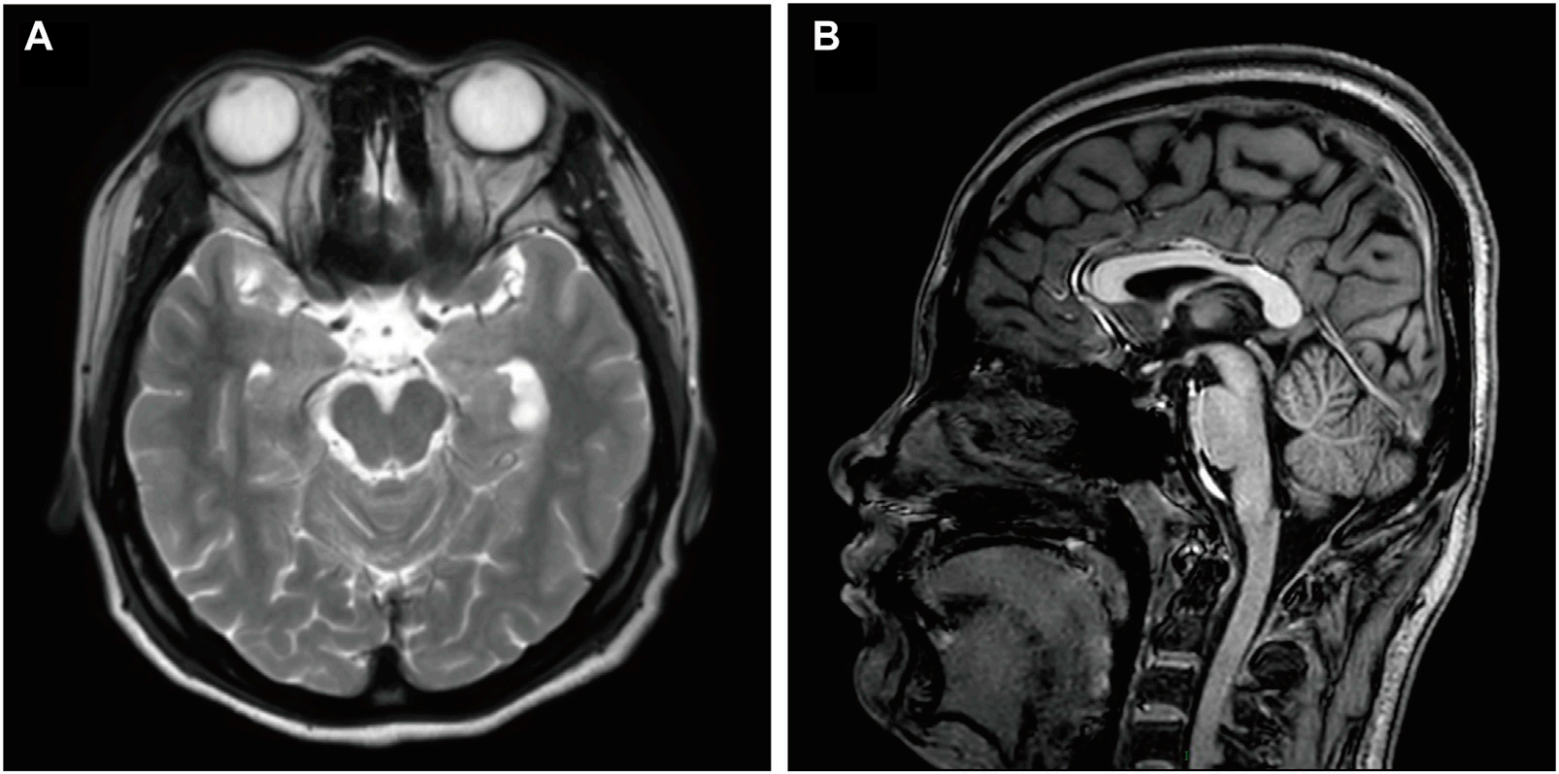
Brain MRI of the described case. **(A)** Axial T2 and **(B)** sagittal T1 brain MRI images of this patient, at an age of 22 years. Image A shows a compressed left hippocampus. Image B shows no cerebellar atrophy. MRI, magnetic resonance imaging; FLAIR, fluid-attenuated inversion recovery.

When the patient was 22 years old, she developed a generalized tremor due to fever, which could not be well controlled with the previous medications. She also walked unsteadily, sometimes stood unsteadily, and had intermittent blurred vision. Complete neurological examination showed generalized tremors, especially in the head and neck region, which were aggravated during activity but decreased at rest. Her limb muscle tone was increased, and ataxia was present. Intellectual disability was also observed. The brain MRI epilepsy sequence showed an abnormal signal near the temporal horn of the left lateral ventricle. A choroid plexus cyst and compression of the left hippocampus were considered **(** Figure 1). This was similar to the MRI findings obtained 8 years before. EEG revealed fast waves and low-to-moderate amplitude slow waves during wakefulness, poor regulation of the amplitude and wave rate, but with abnormal patterns during sleep. Blood and urine tests revealed no lactic acid, pyruvic acid, vitamin B12, thyroid function, ceruloplasmin, copper, or lipoprotein abnormalities. Trio whole-exome sequencing (WES) and mitochondrial gene sequencing were performed, and a novel *NUS1* variant, c.750del (p.Leu251*), was identified. Clonazepam (0.25mg, twice daily) and levetiracetam (500 mg, twice daily) were remarkably effective in controlling the patient’s tremors and unsteady gait.

About 6 months later, the patient presented with whole-body tremors. She was unable to stand upright or walk. Before symptom development, she reported experiencing fatigue for several days. The physical examination results were comparable to those of the previous examinations. Haloperidol (1 mg, twice daily) and levodopa/benserazide (0.0625g, three times a day) were prescribed, which reduced the whole-body tremors. However, 5 months later, the body tremors recurred, sodium valproate and arotinolol hydrochloride were added to the regimen to control the tremors. Unfortunately, the patient continued to exhibit an unsteady gait, slow and slurred communication, and poor numeracy skills, along with deficiencies in logic and comprehension.

### 2.1 Genetic analysis

**FIGURE 2:**
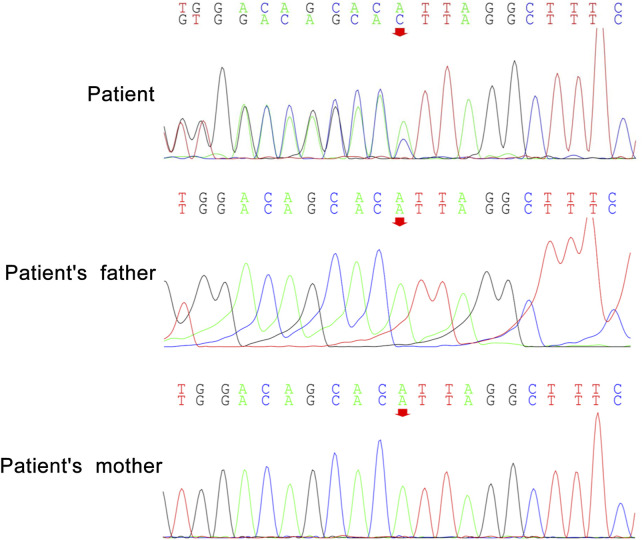
*De novo* heterozygous variants of *NUS1* (c.750del) in our patient.

**FIGURE 3:**
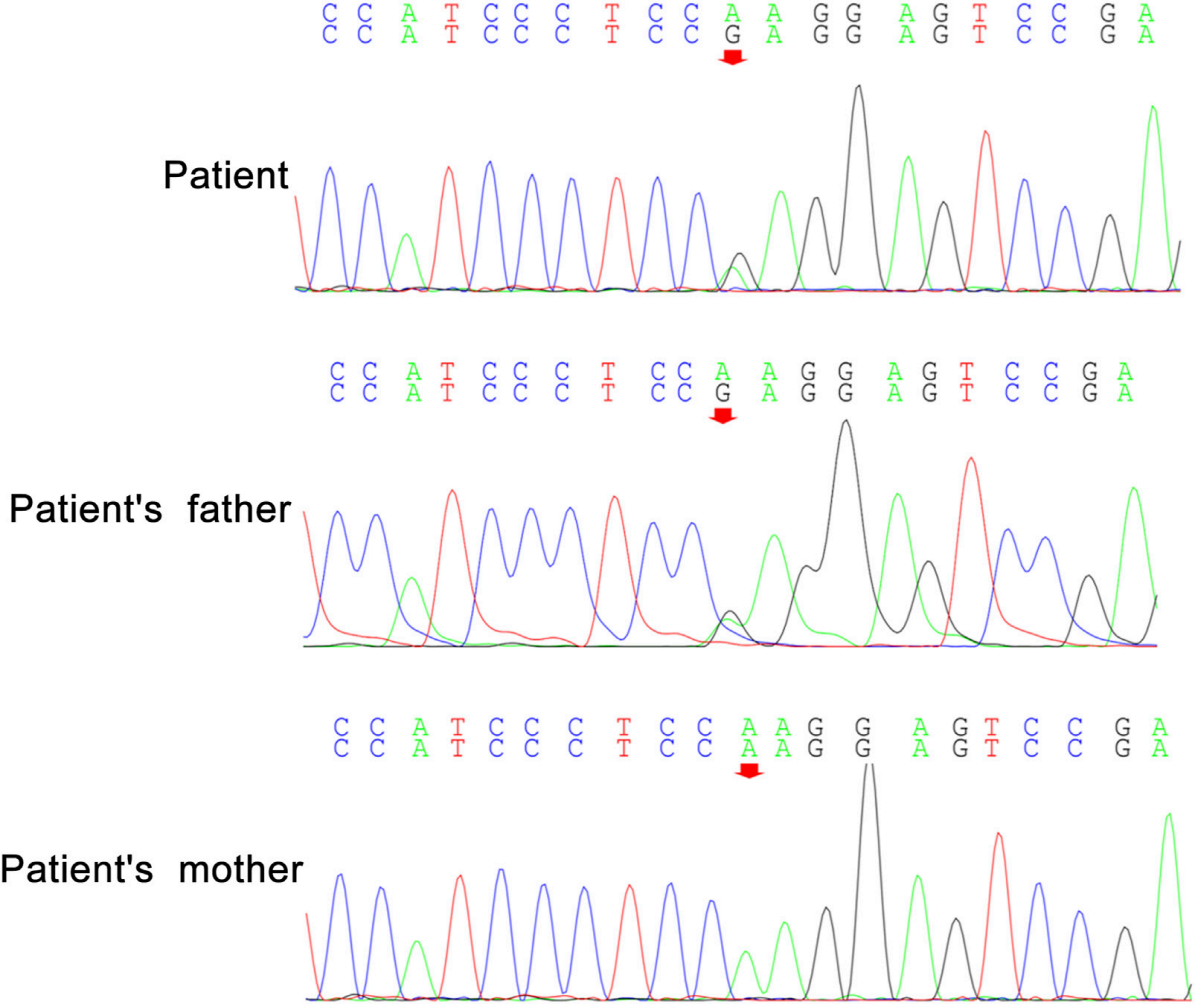
A novel variant c.2026-3A>G of *UBTF* in our patient.

Peripheral blood samples from the patient and her parents were collected for WES and mitochondrial genetic tests to identify the causal gene. WES results revealed a *de novo* variant of *NUS1* (c.750del) (Figure 2) and a novel variant of *UBTF* (c.2026-3A>G) (Figure 3). Mitochondrial gene testing yielded negative results. The c.750del variant of *NUS1* gene was an exon-deletion variant, and was not documented in the NHLBI Exome Sequencing Project, 1000G, and Exome Aggregation Consortium databases. Family-based WES confirmed that the c.750del variant was a *de novo* variant in this case. So according to the pathogenicity classification guidelines of the American College of Medical Genetics and Genomics (ACMG), the c.750del variant of *NUS1* gene is classified as likely pathogenic (very strong evidence of pathogenicity [PVS1], strong evidence of pathogenicity [PS2] and moderate evidence of pathogenicity [PM2]). Moreover, a novel variant (c.2026-3A>G) of the *UBTF* gene on chromosome chr17:42284968 was also identified in our patient. Sequence reads were aligned against the HGMD Pro, PubMed, and ClinVar databases, and this variant had not yet been reported in the medical literature. The *UBTF* gene also revealed a heterozygous variant in the patient’s father. According to the pathogenicity classification guidelines of the ACMG, there is uncertain significance. Although the clinical features (epilepsy and intellectual disability) of our patient matched the phenotype of *UBTF* gene-related diseases, it is noteworthy that her father also carries the same variant without exhibiting any associated phenotype. This observation suggests that either the variant does not have significant clinical implications or that there may be a reduced penetrance of this variant in the family.

## 3 Discussion

The *NUS1* gene is located on chromosome 6q22.1 (NM_138,459.4 and NP_612468.1) and encodes NgBR, with a length of 293 amino acids (Harrison et al., 2011; Szafranski et al., 2015). As a subunit of cis-prenyltransferase, NgBR interacts with DHDDS to promote isoprenyltransferase activity (Harrison et al., 2011; Park et al., 2014; Giladi et al., 2022). The absence of epilepsy and ataxia are more common in the 6q22 mutation than in the 6q16 and 6q21 mutations (Araki et al., 2020). The *NUS1* mutation in 6q22, as a non-ion channel gene, may contribute to the pathogenesis of epilepsy and present as a syndrome characterized by cerebellar ataxia and tremors. Herein, we report a patient presented with tremors, epilepsy, and intellectual disability, attributed to *NUS1* variant.

A novel variant c.750del (p.Leu251*) of *NUS1* (NM_138,459) on chromosome chr6:118024826 was identified in our patient via WES analysis, and it has not been reported in previous cases with heterozygous *NUS1* gene mutations. A novel heterozygous deletion in exon four leads to premature protein termination. The genetic variation site located in the C-terminal structural domain of NgBR is essential for its interaction with the DHDSS. Due to the C-terminal transformation, the NgBR is unable to interact with DHDDS or the substrates, limiting the enzyme activity of the cis-prenyltransferase and negatively affecting its functionality, ultimately leads to tremors, epilepsy, intellectual disability, and ataxia (Harrison et al., 2011). The majority of the pathogenic variants of *NUS1* are severe deleterious mutations (frameshift, stop, splice-disruptive, exon deletions, and chromosomal deletions), resulting in the loss of complete proteins (Araki et al., 2020). The pathogenic features of the identified nonsense mutation consistent with the previously documented haploinsufficiency mechanism associated with this gene (Araki et al., 2020). Further experiments are needed to confirm this observation, and the potential correlation between the genotypic phenotype and the clinical phenotype of this novel variant necessitates further investigation.

Since the first identification of a *NUS1* mutation in 2014, a total of 29 cases with confirmed causative mutations in *NUS1* have been reported, eighteen of which have detailed clinical phenotypes (Park et al., 2014; Riboldi et al., 2022; Hu et al., 2023). *NUS1* variants are rare, and result in various clinical phenotypes, include CDG, MRD55, Parkinson’s disease, dystonia and others (Ji et al., 2023). A comparative analysis was conducted on the symptoms exhibited in 18 previously reported cases of *NUS1* mutations and those observed in our case study, and found varying degrees of overlap in phenotypes. Over half of the cases demonstrated symptoms consistent with those described in our case, including intellectual disability, cerebellar ataxia, tremors, and epileptic seizures. Fever-induced seizures and other symptoms were less common, affecting only two individuals (Hu et al., 2023). The majority of the cases exhibited normal muscular tone, only two patients showed hypotonia. Of note, 84.2% of cases were diagnosed with epilepsy-related disorders, indicating a significant association between *NUS1* variants and epilepsy. In the majority of patients with tremors accompanied by epilepsy and cerebellar ataxia syndrome, MRI has revealed varying degrees of cerebellar atrophy (Den et al., 2019). The brain MRI epilepsy sequences of our patient revealed compression of the left hippocampus. The hippocampus is primarily responsible for short-term memory storage, conversion, and orientation. Indeed, our patient also presented with learning and memory impairment. Although our patient did not show significant cerebellar atrophy on MRI, she did show the clinical phenotype of cerebellar ataxia. This difference may be associated with genetic variations at different gene sites.

Considering the epileptic seizures, intellectual disability, tremors, and identified pathogenic *NUS1* variant, the patient was diagnosed with “congenital glycosylation disorder, type 1aa, autosomal dominant, type 55, with seizures (MRD-55)”, which is a rare congenital disease. It was first reported in three unrelated patients who presented with myoclonic seizures and harbored heterozygous *de novo* pathogenic variants of the *NUS1* gene (Zhang et al., 2021). A study of five familial patients with epilepsy, cerebellar ataxia, and tremors showed a novel heterozygous *NUS1* variant (Hamdan et al., 2017; Zhang et al., 2021). All individuals exhibited cerebellar ataxia and tremors, with three presented with intellectual disabilities but no seizures, one showed generalized epilepsy, and one presented parkinsonism without epilepsy.

Based on previous reports on the treatment of patients with *NUS1* gene mutations, oxcarbazepine has been shown to improve speech and motor functions. Furthermore, baclofen demonstrated a notable reduction in myoclonus and a modest improvement in gait disturbances in the patient (Den et al., 2019; Zhang et al., 2021). Valproate has demonstrated efficacy in managing epilepsy and tremors in patients with *NUS1* mutations in comparison with a control (Haginoya et al., 2021). In a patient who presented with bradykinesia and resting tremors, zonisamide treatment effectively controlled the symptoms; nevertheless, the symptoms were levodopa-resistant (Araki et al., 2020). Our patient was administered with levetiracetam, clonazepam, levodopa, and benserazide hydrochloride. Her symptoms were alleviated and effectively controlled throughout the follow-up phase.

At present, the prevalence of cases exhibiting *NUS1* pathogenic variants remains limited, and the absence of thorough documentation and characterization for a substantial proportion of these cases, hinders the delineation of a definitive genotype-phenotype correlation, especially given the wide spectrum of diseases associated with *NUS1* pathogenic variants. Otherwise, a clear pattern of drug efficacy is not apparent in the cases that underwent drug treatment. The precise molecular mechanisms by which *NUS1* pathogenic variants contribute to the manifestation of various diseases in affected patients remain unclear. Additional in-depth case studies are necessary to clarify the potential correlation between *NUS1* variants and disease progression. Further investigation and functional analyses are required to improve understanding of the *NUS1* gene and its related disorders.

## Data Availability

The original contributions presented in the study are included in the article/Supplementary Materials, further inquiries can be directed to the corresponding author.
